# A Short and Efficient Total Synthesis of Ficuseptamines A and B

**DOI:** 10.3390/molecules23081865

**Published:** 2018-07-26

**Authors:** Hani Mutlak A. Hassan

**Affiliations:** King Fahd Medical Research Center, King Abdulaziz University, P.O. Box 80216, Jeddah 21589, Saudi Arabia; hmahassan@kau.edu.sa

**Keywords:** ficuseptamines A and B, cross metathesis, total synthesis

## Abstract

A rapid and efficient total synthesis of ficuseptamines A and B by a cross metathesis strategy is described.

## 1. Introduction

**Figure 1:**
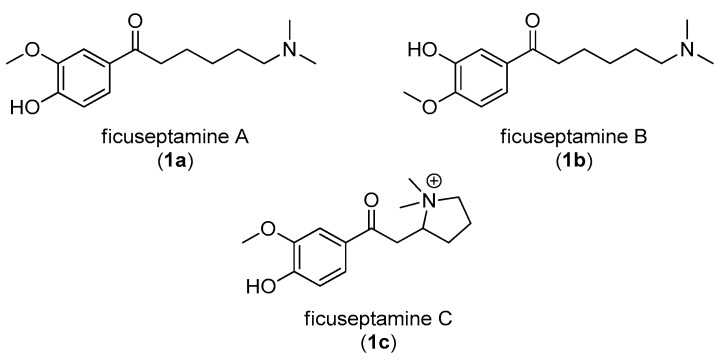
Structures of ficuseptamine A, B, and C (**1a**–**c**).

Ficuseptamines A, B, and C (**1a**–**c**) were isolated from the leaves of *Ficus septica* and reported by Shin-ya and co-workers in 2009 (Figure 1) [1]. Ficuseptamines A and B possess an aminocaprophenone structure, while ficuseptamine C contains a pyrrolidine moiety in its structure. After their isolation, these alkaloids were evaluated for cytotoxicity against HeLa (human cervical carcinoma) and ACC-MESO-1 (malignant pleural mesothelioma) cancer cell lines. Ficuseptamine A displayed IC_50_ values of 57 μM and 160 μM against HeLa and ACC-MESO-1 cells lines, respectively. Ficuseptamine B showed better cytotoxicity against the same cell lines (IC_50_: 23 μM for HeLa; 72 μM for ACC-MESO-1), while ficuseptamine C showed no activity against either cell line. The aryl ketone motif, which is present in ficuseptamines A and B, is frequently found in natural products [2,3] and biologically active molecules [4]. We were interested in devising a novel strategy targeting ficuseptamines A and B for their first total synthesis. We thought that designing an efficient and facile strategy for their rapid total synthesis would offer the possibility of synthesizing ficuseptamine A and B analogues for biological evaluation, given the commercial availability of a wide variety of functionalized terminal olefins, which could be utilized in library design and synthesis. In addition, the incorporation of fluorine atom(s) into ficuseptamines A and B to synthesize fluorinated analogues could also significantly improve their biological activity [5,6].

**Figure 2:**
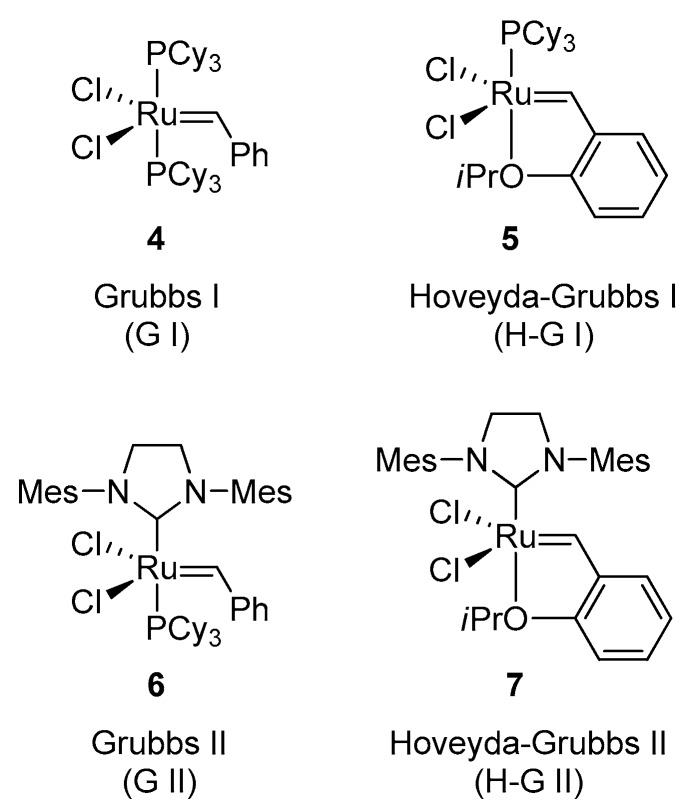
Ruthenium-based olefin metathesis catalysts.

Olefin metathesis is one of the most useful carbon–carbon bond-forming reactions, and it has found tremendous use in organic chemistry for the construction of a myriad of organic molecules [7,8,9,10]. This highly powerful transformation has also been elegantly utilized in the total synthesis of numerous natural products [11,12]. Olefin metathesis catalysts that promote this transformation are displayed in Figure 2. Cross metathesis (CM) is one of the most popular transformations for connecting two independent olefins together to form a more complex olefinic product, which could require several steps to synthesize if a different methodology was employed. Although ring-closing metathesis (RCM) has found extensive use in the total synthesis of numerous natural products, CM has become increasingly popular in the field of total synthesis [13,14,15], particularly after the discovery of *Z*-selective olefin metathesis catalysts [16]. As part of our interest in the power of olefin metathesis to facilitate useful transformations [17], we report herein a highly efficient methodology for the total synthesis of ficuseptamines A and B, utilizing a cross metathesis approach.

## 2. Results and Discussion

**Scheme 1:**
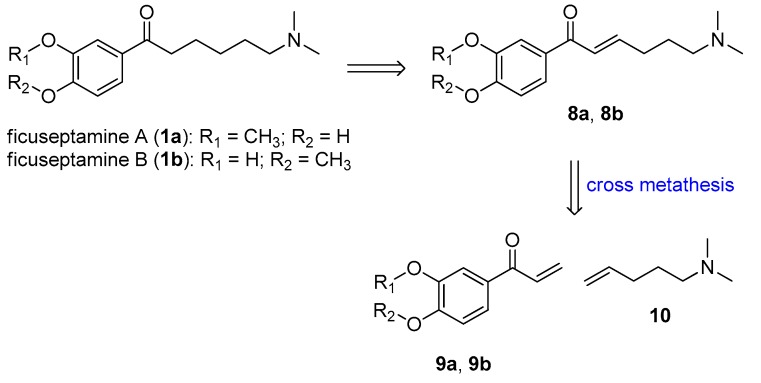
Retrosynthetic analysis of ficuseptamines A and B.

**Scheme 2:**
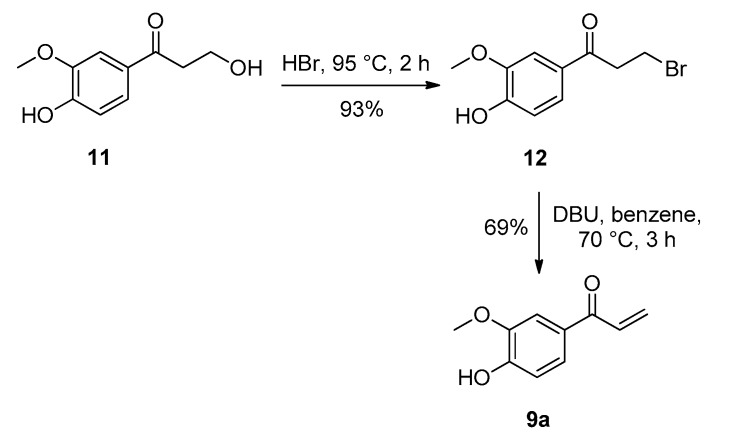
Bromination of **11** followed by dehydrobromination with DBU to give CM precursor **9a**.

A retrosynthetic analysis of ficuseptamines A and B is depicted in Scheme 1. We commenced the synthetic work by first targeting ficuseptamine A for total synthesis. Aryl ketone **9a** was prepared in two sequential steps from commercially available alcohol **11** via halogenation and dehydrohalogenation reactions (Scheme 2).

Thus, primary alcohol **11** was transformed into its bromo counterpart (i.e., **12**), under the influence of hydrobromic acid to furnish **12** in high yield (93%). Subsequent treatment of the brominated product **12** with DBU (1,8-diazabicyclo[5.4.0]undec-7-ene) delivered CM precursor **9a** in 69% yield through dehydrobromination.

Next, we explored the key CM reaction between **9a** and *N*,*N*-dimethyl-4-pentene-1-amine **10** to afford the α,β-unsaturated CM product **8a**. We initially performed the CM reaction using Grubbs I catalyst **4** (5 mol %) with CH_2_Cl_2_ as a solvent to afford CM product **8a** in 22% yield (entry 1, Table 1). Increasing the Grubbs I catalyst **4** loading (10 mol %) provided **8a** in a disappointing 15% yield (entry 2, Table 1). We then turned our attention to Grubbs II catalyst **5**, which gave the CM product **8a** in modest 37 and 41% yields using 5 mol % and 10 mol % catalyst loading in CH_2_Cl_2_, respectively (entries 3 and 4, Table 1). Switching the solvent from CH_2_Cl_2_ to toluene and performing the reaction with Grubbs II catalyst **5** (10 mol %) at 80 °C improved the yield to 47% (entry 5, Table 1).

A report by Grubbs and co-workers described how the presence of the phosphine ligand (PCy_3_) in olefin metathesis catalysts can attack the carbine through its dissociation from the metal complex via decomposition [18]. This fact turned our attention to Hoveyda-Grubbs II **7**, which lacks the PCy_3_ ligand. Pleasingly, the use of Hoveyda-Grubbs II **7** (5 mol %) increased the yield significantly, to afford **8a** in a 68% yield using CH_2_Cl_2_ as the solvent (entry 6, Table 1). Treating **9a** and **10** with an increased Hoveyda-Grubbs II **7** loading (10 mol %) in CH_2_Cl_2_ provided **8a** in an excellent 76% yield (entry 7, Table 1). In contrast, increasing the temperature to 80 °C and changing the solvent from CH_2_Cl_2_ to toluene diminished the yield to 52% (compare entry 7 vs. 8, Table 1). The double bond geometry of the CM product **8a** was identified as the (*E*)-configured isomer, indicating that CM proceeded selectively to give the thermodynamically stable (*E*)-isomer exclusively. A change in molar ratio of the coupling partners **9a** and **10,** and performing the reaction using 10 mol % of Hoveyda-Grubbs II **7** in CH_2_Cl_2_, resulted in a reduction of the yield to 42%, with formation of the homodimer of **10** as a competing side-product (entry 9, Table 1). Grubbs and co-workers categorized olefin metathesis substrates as Type I, Type II, Type III, or Type IV [19]. According to Grubbs’ selectivity model, terminal olefin **10** is a Type I olefin substrate, which usually undergoes fast homodimerization, while olefin **9a** is a Type II olefin, which undergoes slow homodimerization. Thus, such a change in the ratio of the coupling partners **9a** and **10** has promoted homodimerization of **10** to occur.

**Scheme 3:**
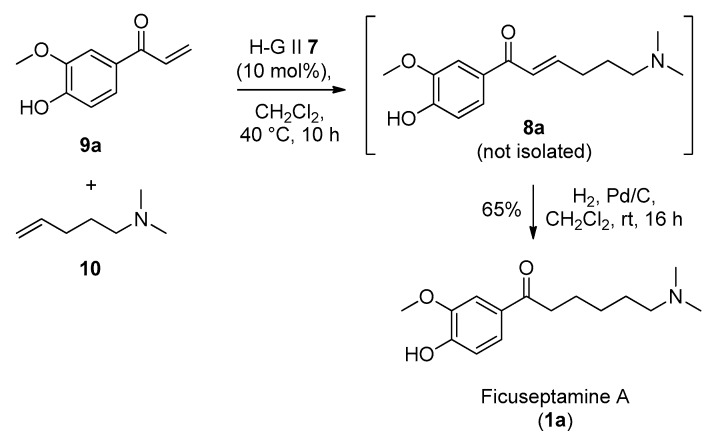
Improving ficuseptamine A (**1a**) synthesis by a one-pot CM/hydrogentation sequence.

With synthesis of compound **8a** accomplished through CM, saturation of the newly formed double bond (Pd/C, H_2_, CH_3_OH, r.t., 16 h) proceeded smoothly to provide ficuseptamine A in 62% isolated yield. We then envisioned performing one-pot CM/hydrogenation reactions for the synthesis of ficuseptamine A without isolation of the unsaturated CM product **8a**. In fact, a literature screen revealed that such a CM/hydrogenation maneuver had been reported by Cossy and co-workers in their total synthesis of (‒)-centrolobine [20]. One-pot sequential reactions are increasingly being utilized in organic synthesis to accelerate the synthesis of target molecules [21]. Motivated by Cossy’s work, we subjected aryl ketone **9a** and terminal olefin **10** to our optimized CM conditions, followed by hydrogenation in a one-pot fashion, to afford ficuspetamine A in 65% yield directly from the starting materials **9a** and **10** (Scheme 3). This one-pot CM/hydrogenation sequence proved to be fruitful, thus improving the efficiency of the synthesis of ficuseptamine A.

**Scheme 4:**
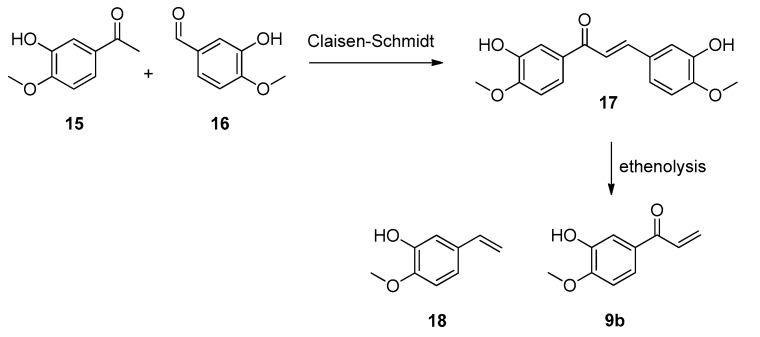
Synthetic plan for the synthesis of aryl ketone **9b** via a Claisen-Schmidt reaction followed by ethenolysis.

With ficuseptamine A (**1a**) synthesis accomplished, we moved forward to target ficuseptamine B (**1b**). We imagined employing a dual ethenolysis [22] and CM approach for its total synthesis. We envisaged that aryl ketone **9b**, which is a different regioisomer to aryl ketone **9a**, could be achieved by ethenolysis of the Claisen-Schmidt product **17**, using a suitable olefin metathesis catalyst to dissect the internal alkene of **17** to give the desired terminal olefin **9b** (Scheme 4). We also thought that this method would allow access to styrene derivatives (e.g., compounds such as **18**), which are important building blocks in organic chemistry.

We began the synthesis towards ficuseptamine B by first preparing the Claisen-Schmidt product **17**. We initially combined 3-hydroxy-4-methoxyacetophenone **15** and 3-hydroxy-4-methoxybenzaldehyde **16** under aqueous NaOH conditions in EtOH, which provided chalcone **17** in 46% yield (entry 1, Table 2). The use of cesium carbonate (Cs_2_CO_3_) as the base in EtOH led to **17** in 41% yield (entry 2, Table 2). However, efficient synthesis of **17** was achieved using SOCl_2_/EtOH as a catalyst system (acid catalysis) [23] to afford **17** in 83% yield (entry 3, Table 2).

**Scheme 5:**
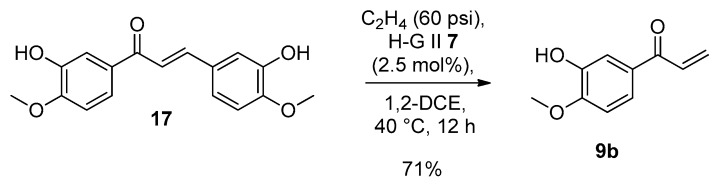
Ethenolysis of chalcone **17** to deliver aryl ketone **9b**.

With the Claisen-Schmidt product **17** in hand, we employed the conditions reported by Diver and co-workers with slight modification for its ethenolysis [24]. Exposing **17** to ethylene gas (60 psi pressure) with Hoveyda-Grubbs II **7** and using 1,2-DCE (1,2-dichloroethane) as a solvent at 40 °C delivered aryl ketone **9b** in 71% yield (Scheme 5).

**Scheme 6:**
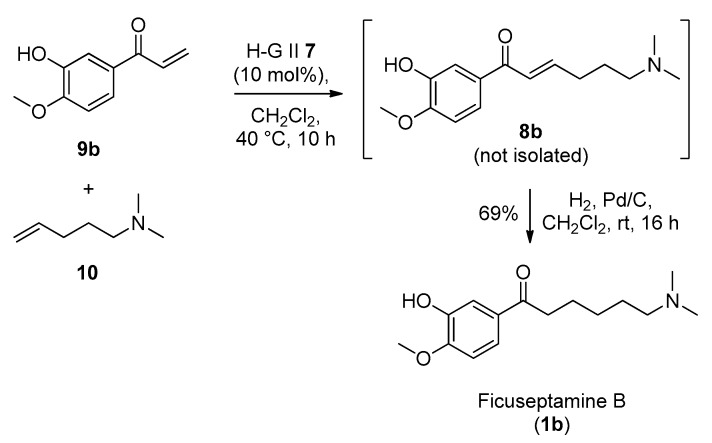
Synthesis of ficuseptamine B (**1b**) by one-pot CM/hydrogenation sequence.

To the best of our knowledge, this is the first time ethenolysis has been applied to cleave a chalcone compound (i.e., structure such as **17**). Importantly, no isomerization [25] of the double bond is possible, given the structure of chalcone **17,** which lacks any methylene groups adjacent to the double bond. Having accomplished the synthesis of aryl ketone **9a** through ethenolysis, we employed the one-pot, two-step CM/hydrogenation protocol previously used in ficuseptamine A synthesis. Gratifyingly, subjecting aryl ketone **9b** and terminal olefin **10** to our optimized CM conditions (Hoveyda-Grubbs II **7** (10 mol %), CH_2_Cl_2_, 40 °C) followed by one-pot hydrogenation delivered ficuseptamine B in 69% yield (Scheme 6).

The spectroscopic data of the synthetic ficuseptamines A and B were identical to those reported by Shin-ya and co-workers [1]. The described chemistry for the synthesis of ficuseptamines A and B through CM allows the generation of various analogues of these natural products in a straightforward manner, which could have other biological activities, such as potential ligands for the 5-HT_7_ receptor [26].

## 3. Conclusions

In summary, we have reported a highly efficient methodology for the first total synthesis of ficuseptamines A and B through a CM strategy. A sequential one-pot, two-step CM/hydrogenation procedure was employed to expediently accomplish their total synthesis.

## 4. Materials and Methods

### 4.1. General Chemistry Experimental

Chemical reactions were performed in over-dried glassware under nitrogen and anhydrous conditions, unless otherwise stated. Reactions were magnetically stirred using a Teflon-coated stir bar and monitored by pre-coated silica gel aluminum plates (0.25 mm thickness) with a fluorescent indicator (254 nm), using UV light as the visualizing agent. Alternatively, oxidative staining using an aqueous basic solution of KMnO_4_ and heat was carried out for visualization. Silica gel (60 Å, 200–425 mesh) was used for flash column chromatography. Nuclear magnetic resonance (NMR) spectra were recorded on a Bruker 400 MHz spectrometer (Bruker, Billerica, MA, USA) in acetone-*d*_6_, CDCl_3_, or DMSO-*d*_6_ as the solvent. Chemical shifts are reported in parts per million (ppm) with reference to the hydrogenated residues of the deuterated solvent as the internal standard. Coupling constants (*J* values) are recorded in Hertz (Hz), and signal patterns are expressed as follows: singlet (s), doublet (d), dd (doublet of doublets), triplet (t), quintet (quint), and multiplet (m). Elemental analyses were performed on a 2400 Perkin Elmer Series II analyzer (PerkinElmer, Inc., Waltham, MA, USA). High-resolution mass spectrometry was conducted using a Micromass Q-ToF mass spectrometer (Waters Corporation, Milford, MA, USA).

### 4.2. Experimental Procedures for Chemical Synthesis and Characterization Data of Compounds

#### 4.2.1. Synthesis of 1-(4-Hydroxy-3-methoxyphenyl)prop-2-en-1-one (**9a**)

A round-bottom flask equipped with a magnetic stir bar was charged with 3-hydroxy-1-(4-hydroxy-3-methoxyphenyl)propan-1-one **11** (2.52 g, 12.8 mmol) and conc. HBr (30 mL) was then added. The reaction mixture was then stirred at 95 °C for 2 h. The reaction mixture was cooled to room temperature and the resulting precipitate was filtered, washed with ice-cold H_2_O, and dried to afford 3-bromo-1-(4-hydroxy-3-methoxyphenyl)propan-1-one **12** (quantitative yield, 3.09 g, 93%) as a white solid, which was judged to be of good purity by TLC analysis and mass spectrometry, and carried forward in crude form to the next step. To a stirred solution of 3-bromo-1-(4-hydroxy-3-methoxyphenyl)propan-1-one **12** (3 g, 11.6 mmol) in dry benzene (50 mL), was added, dropwise, a solution of DBU (2.08 mL, 13.9 mmol) in dry benzene (5 mL). A condenser was attached, and the reaction mixture was stirred at 70 °C for 3 h. The solvent was removed in vacuo, and the crude product was extracted with EtOAc (three times). The combined organic layers were washed with H_2_O, brine, dried over MgSO_4_, filtered, and concentrated in vacuo. The crude product was then purified by silica gel column chromatography to afford **9a** (1.42 g, 69%) as a white solid. ^1^H-NMR (400 MHz, acetone-*d*_6_): δ 7.66 (dd, *J* = 8.4, 2.0 Hz, 1H), 7.61 (d, *J* = 2.0 Hz, 1H), 7.40 (dd, *J* = 17.0, 10.4 Hz, 1H), 6.95 (d, *J* = 8.4 Hz, 1H), 6.36 (dd, *J* = 17.0, 2.0 Hz, 1H), 5.86 (dd, *J* = 10.4, 2.0 Hz, 1H), 3.93 (s, 3H); HRMS calcd. for C_10_H_11_O_3_ [M + H]^+^ 179.0708, found 179.0713. Anal. calcd. for C_10_H_10_O_3_: C, 67.41; H, 5.66. Found: C, 67.31; H, 5.55.

#### 4.2.2. Synthesis of Ficuseptamine A by a One-Pot Cross Metathesis/Hydrogenation Procedure (**1a**)

To a stirred solution of aryl ketone **9a** (315 mg, 1.77 mmol) and *N*,*N*-dimethyl-4-pentene-1-amine **10** (100 mg, 0.885 mmol) in dry and degassed CH_2_Cl_2_ (5 mL), was added Hoveyda-Grubbs second generation catalyst **7** (55 mg, 0.0885 mmol, 10 mol %). The reaction mixture was then deoxygentated by performing vacuum/N_2_ cycles four times and stirred at 40 °C for 10 h. After the completion of the reaction, Pd/C (10 wt %) was added, and the N_2_ atmosphere was substituted with H_2_ by performing vacuum/H_2_ cycles four times. The reaction mixture was then stirred under a double layer H_2_ balloon at 23 °C for 16 h. The Pd/C catalyst was removed by filtration through a pad of Celite^®^, followed by washing of the filter cake with CH_2_Cl_2_ (10 mL), and the filtrate was evaporated in vacuo. The crude product was purified directly by silica gel column chromatography to afford ficuseptamine A (**1a**) as a white solid (153 mg, 65%). Spectroscopic data for synthetic ficuseptamine A matched literature data of natural ficuseptamine A [1]. ^1^H-NMR (400 MHz, acetone-*d*_6_): δ 7.57 (dd, *J* = 8.4, 2.0 Hz, 1H), 7.54 (d, *J* = 2.0 Hz, 1H), 6.89 (d, *J* = 8.4 Hz, 1H), 3.89 (s, 3H), 2.93 (t, *J* = 7.2 Hz, 2H), 2.21 (t, *J* = 7.2 Hz, 2H), 2.13 (s, 2 × 3H), 1.68 (quint, *J* = 7.2 Hz, 2H), 1.47 (quint, *J* = 7.2 Hz, 2H), 1.37 (quint, *J* = 7.2 Hz, 2H); ^13^C-NMR (100 MHz, acetone-*d*_6_) δ: 198.6, 152.1, 148.3, 130.6, 123.8, 115.3, 111.5, 60.2, 56.2, 45.6, 38.4, 28.3, 27.8, 25.3; HRMS calcd. for C_15_H_24_NO_3_ [M + H]^+^ 266.1756, found 266.1767. Anal. calcd. for C_15_H_23_NO_3_: C, 67.90; H, 8.74; N, 5.18. Found: C, 67.76; H, 8.70; N, 5.07.

#### 4.2.3. Synthesis of (2*E*)-1,3-Bis(3-Hydroxy-4-methoxyphenyl)prop-2-en-1-one (**17**)

Compound **17** was synthesized using a previously reported method, except that it required purification [23]. To a stirred solution of 3-hydroxy-4-methoxyacetophenone **15** (1.20 g, 7.89 mmol) and 3-hydroxy-4-methoxybenzaldehyde **16** (1.31 g, 7.89 mmol) in absolute EtOH (5 mL), was added thionyl chloride (0.58 mL, 7.89 mmol) dropwise and the reaction mixture was stirred at 23 °C for 4 h. The reaction mixture was precipitated by the addition of H_2_O (~5 mL), and the resulting precipitate was filtered, washed with ice-cold H_2_O, and ice-cold EtOH. After the crude product was allowed to air dry, purification by recrystallization from EtOH afforded **17** (1.97 g, 83%) as a yellow solid. Analytical data were in accordance with literature data [27]. ^1^H-NMR (400 MHz, CDCl_3_): δ 7.72 (d, *J* = 16.2 Hz, 1H), 7.63-7.60 (m, 2H), 7.40 (d, *J* = 16.2 Hz, 1H), 7.28-7.27 (m, 1H), 7.12 (dd, *J* = 10.4, 2.0 Hz, 1H), 6.93 (d, *J* = 8.2 Hz, 1H), 6.85 (d, *J* = 8.2 Hz, 1H), 3.98 (s, 3H), 3.94 (s, 3H); ^13^C NMR (100 MHz, DMSO-*d*_6_): δ 188.5, 152.7, 150.7, 147.0, 146.9, 144.3, 131.3, 128.1, 122.8, 122.4, 119.8, 115.2, 114.8, 112.5, 111.8, 56.3, 56.2; HRMS calcd. for C_17_H_17_O_5_ [M + H]^+^ 301.1076, found 301.1065. Anal. calcd. for C_17_H_16_O_5_: C, 67.99; H, 5.37. Found: C, 67.80; H, 5.41.

#### 4.2.4. Synthesis of 1-(3-Hydroxy-4-methoxyphenyl)prop-2-en-1-one (**9b**)

Compound **9b** was synthesized using the slightly modified reaction conditions of Diver et al. [24]. To an inert and oven-dried high-pressure flask, was added compound **17** (800 mg, 2.67 mmol), dry and degassed 1,2-dichloroethane (35 mL), and Hoveyda-Grubbs second generation catalyst **7** (42 mg, 0.067 mmol). The flask was purged with ethylene for 5 min, pressurized to 60 psi, and stirred at 40 °C for 12 h, after which time TLC analysis indicated consumption of the internal alkene **17**. The pressure was then slowly vented, and the solvent was evaporated in vacuo. The crude product was then purified directly by silica gel column chromatography, to afford **9b** (337 mg, 71%) as a white solid. ^1^H-NMR (400 MHz, acetone-*d*_6_): δ 7.59 (dd, *J* = 8.4, 2.0 Hz, 1H), 7.50 (d, *J* = 2.0 Hz, 1H), 7.33 (dd, *J* = 17.0, 10.4 Hz, 1H), 7.07 (d, *J* = 8.4 Hz, 1H), 6.33 (dd, *J* = 17.0, 2.0 Hz, 1H), 5.84 (dd, *J* = 10.4, 2.0 Hz, 1H), 3.94 (s, 3H); ^13^C NMR (100 MHz, acetone-*d*_6_): δ 188.9, 153.0, 147.7, 133.2, 131.8 128.9, 122.8, 115.7, 111.8, 56.5; HRMS calcd. for C_10_H_11_O_3_ [M + H]^+^ 179.0708, found 179.0719. Anal. calcd. for C_10_H_10_O_3_: C, 67.41; H, 5.66. Found: C, 67.35; H, 5.59.

#### 4.2.5. Synthesis of Ficuseptamine B by a One-Pot Cross Metathesis/Hydrogenation Procedure (**1b**)

The experimental procedure for the synthesis of ficuseptamine A (**1a**) was followed, except that aryl ketone **9b** was used as the cross metathesis partner with *N*,*N*-dimethyl-4-pentene-1-amine **10** to afford ficuspetamine B (**1b**) (162 mg, 69%) as a white solid. Spectroscopic data for synthetic ficuseptamine B matched literature data of natural ficuseptamine B [1]. ^1^H-NMR (400 MHz, acetone-*d*_6_): δ 7.52 (dd, *J* = 8.5, 2.0 Hz, 1H), 7.46 (d, *J* = 2.0 Hz, 1H), 7.01 (d, *J* = 8.5 Hz, 1H), 3.90 (s, 3H), 2.90 (t, *J* = 7.2 Hz, 2H), 2.24 (t, *J* = 7.2 Hz, 2H), 2.15 (s, 2 × 3H), 1.66 (quint, *J* = 7.2 Hz, 2H), 1.48 (quint, *J* = 7.2 Hz, 2H), 1.37 (quint, *J* = 7.2 Hz, 2H); ^13^C-NMR (100 MHz, acetone-*d*_6_): δ 198.9, 152.4, 147.3, 131.7, 121.7, 115.2, 111.5, 60.1, 56.3, 45.5, 38.5, 28.1, 27.7, 25.2; HRMS calcd. for C_15_H_24_NO_3_ [M + H]^+^ 266.1756, found 266.1741. Anal. calcd. for C_15_H_23_NO_3_: C, 67.90; H, 8.74; N, 5.28. Found: C, 67.78; H, 8.66; N, 5.19.

## Figures and Tables

**Table 1:**
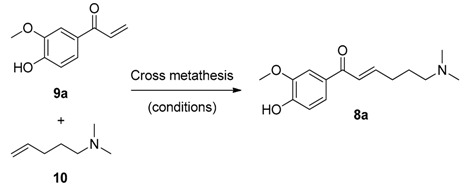
Optimization of the CM reaction.

E	Substrate 9a (equiv.)	Substrate 10 (equiv.)	Catalyst (Loading)	Solvent	T (°C)	Yield % ^a^
1	2	1	G I **4** (5 mol %)	CH_2_Cl_2_	40	22
2	2	1	G I **4** (10 mol %)	CH_2_Cl_2_	40	15
3	2	1	G II **5** (5 mol %)	CH_2_Cl_2_	40	37
4	2	1	G II **5** (10 mol %)	CH_2_Cl_2_	40	41
5	2	1	G II **5** (10 mol %)	Toluene	80	47
6	2	1	H-G II **7** (5 mol %)	CH_2_Cl_2_	40	68
7	2	1	H-G II **7** (10 mol %)	CH_2_Cl_2_	40	76
8	2	1	H-G II **7** (10 mol %)	Toluene	80	52
9	1	2	H-G II **7** (10 mol %)	CH_2_Cl_2_	40	42

^a^ All reactions were performed using **9a** (2 mmol), **10** (1 mmol), solvent, and temperature for 10 h, except entry 9, in which the reaction was carried out using **9a** (1 mmol) and **10** (2 mmol). E = entry.

**Table 2:**
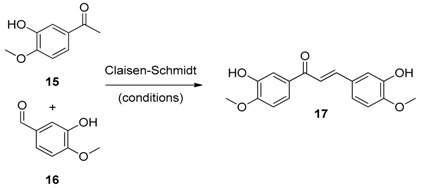
Synthesis of Claisen-Schmidt product **17** under various catalysis conditions. ^a^ All reactions were carried out using ketone **15** (1 equiv.), aldehyde **16** (1 equiv.), and catalyst in EtOH at 23 °C. ^b^ NaOH (10% *w*/*v*).

Entry	Catalyst	Equivalent	Time (h)	Yield (%) ^a^
1	NaOH ^b^	2	16	46
2	Cs_2_CO_3_	2	16	41
3	SOCl_2_	1	4	83
